# Fetal aortic isthmus Doppler assessment to predict the adverse perinatal outcomes associated with fetal growth restriction: systematic review and meta-analysis

**DOI:** 10.1007/s00404-023-06963-4

**Published:** 2023-04-19

**Authors:** M. La Verde, F. Savoia, G. Riemma, A. Schiattarella, A. Conte, S. Hidar, M. Torella, N. Colacurci, P. De Franciscis, M. Morlando

**Affiliations:** 1 Department of Woman, Child and General and Specialized Surgery, Obstetrics and Gynecology Unit, University of Campania “Luigi Vanvitelli”, Largo Madonna delle Grazie 1, 80138 Naples, Italy; 2 Obstetrics and Gynecology Department, F. Hached University Teaching Hospital, 4000 Sousse, Tunisia

**Keywords:** Fetus, Aortic isthmus, Fetal growth retardation, IUGR, Doppler

## Abstract

**Purpose:**

Fetal growth restriction (FGR) management and delivery planning is based on a multimodal approach. This meta-analysis aimed to evaluate the prognostic accuracies of the aortic isthmus Doppler to predict adverse perinatal outcomes in singleton pregnancies with FGR.

**Methods:**

PubMed, EMBASE, the Cochrane Library, ClinicalTrials.gov and Google scholar were searched from inception to May 2021, for studies on the prognostic accuracy of anterograde aortic isthmus flow compared with retrograde aortic isthmus flow in singleton pregnancy with FGR. The meta-analysis was registered on PROSPERO and was assessed according to PRISMA and Newcastle–Ottawa Scale. DerSimonian and Laird’s random-effect model was used for relative risks, Freeman-Tukey Double Arcsine for pooled estimates and exact method to stabilize variances and CIs. Heterogeneity was quantified using I^2^ statistics.

**Results:**

A total of 2933 articles were identified through the electronic search, of which 6 studies (involving 240 women) were included. The quality evaluation of studies revealed an overall acceptable score for study group selection and comparability and substantial heterogeneity. The risk of perinatal death was significantly greater in fetuses with retrograde Aortic Isthmus blood flow, with a RR of 5.17 (*p* value 0.00001). Similarly, the stillbirth rate was found to have a RR of 5.39 (*p* value 0.00001). Respiratory distress syndrome had a RR of 2.64 (*p* value = 0.03) in the group of fetuses with retrograde Aortic Isthmus blood flow.

**Conclusion:**

Aortic Isthmus Doppler study may add information for FGR management. However, additional clinical trial are required to assess its applicability in clinical practice.

**Supplementary Information:**

The online version contains supplementary material available at 10.1007/s00404-023-06963-4.

## What does this study add to the clinical work

**Table:** 

Aortic isthmus Doppler represents an addionatl doppler marker useful for FGR management. However, its role is not entirely known, and our meta-analysis demonstrated that the aortic isthmus Doppler study could predict neonatal adverse outcomes.	

## Introduction

Fetal growth restriction (FGR) occurs in around 10% of gestations and represents a significant cause of perinatal morbidity and mortality [1, 2]. The stillbirth incidence is twice (1.5%) in these fetuses than in fetuses with normal growth [3, 4]. FGR is a complicated obstetrical dilemma with different queries, such as low detection rates or few prophylactic interventions. FGR can be classified as early-onset or late-onset based on gestational age at prenatal ultrasound diagnosis. FGR classification had several implications for diagnosis, treatment and prognosis. Early-onset FGR is diagnosed before 32 weeks of gestation [5]. This type of FGR is usually severe, follows a well-established Doppler deterioration pattern, and is frequently correlated with hypertensive disorders of pregnancy [2]. Late-onset FGR is diagnosed after 32 weeks of gestation and is correlated with placental insufficiency and chronic intrauterine hypoxia more frequently than gestational hypertensive disorders. Once early-onset or late-onset FGR has been diagnosed, further evaluation and monitoring are required to determine the optimal delivery timing [5, 6]. Management also provides several criticisms. Different management algorithms have been proposed, involving the use of ultrasound (US) and cardiotocography evaluations [5, 7–14]. Umbilical artery (UA) Doppler has been proven to be an essential surveillance tool, mainly in the presence of an absent or reversed end-diastolic flow (EDF) [7, 15–17]. Additional Doppler parameters as the fetal middle cerebral artery (MCA), the cerebroplacental ratio (CPR) and the ductus venosus (DV) are progressively integrated into the FGR management [10, 18–22]. Altered MCA and CPR are considered expression of the “brain sparing effect”, a signal of fetal circulation redistribution [23–26]. The correlation between MCA Doppler and the adverse fetal outcome has been described [27, 28] and the same was reported for an abnormal CPR [29, 30]. Uterine artery Doppler (UtA) abnormalities have also been associated with the occurrence of stillbirth and adverse perinatal outcomes [31–33]. However, as the risks associated with iatrogenic prematurity are very high before 32 weeks’ gestation, additional Doppler parameters are needed to assess fetal compromise and to indicate delivery. According to this, before 32 weeks’ gestation, the main sign of fetal distress to be considered is the presence of abnormal venous flow findings, including reversed flow in the DV during atrial contraction. However, the recognition of such venous alterations is frequently associated with advanced fetal compromise and therefore signs of acidemia and cardiac decompensation. The aortic isthmus (AoI) Doppler has also been studied in fetuses with FGR, as a potential indicator of worsening of the fetal hemodynamic state [34–40]. AoI provides information on fetal haemodynamic circulation, mainly on the ventricular performance [41]. A FGR fetus with normal anterograde flow in AoI, provides a preferential supply of oxygenated blood to the coronary and cerebral circulation [42]. Otherwise, a predominant reverse diastolic blood flow through the AoI, led to a significant decrease of oxygen supply to the brain [43, 44]. Interestingly, the AoI Doppler abnormalities were reported to occur prior to ductus venosus, suggesting that reversed aortic isthmus flow may represent an intermediate step between placental insufficiency-hypoxemia and cardiac failure, a further step in the sequence of Doppler deterioration beginning with the UA and MCA Dopplers [45]. On these considerations, we provided a systematic review and meta-analysis of studies comparing anterograde to retrograde AoI flow as a predictor of adverse perinatal outcomes in fetuses with FGR.

## Materials and methods

**Fig. 1:**
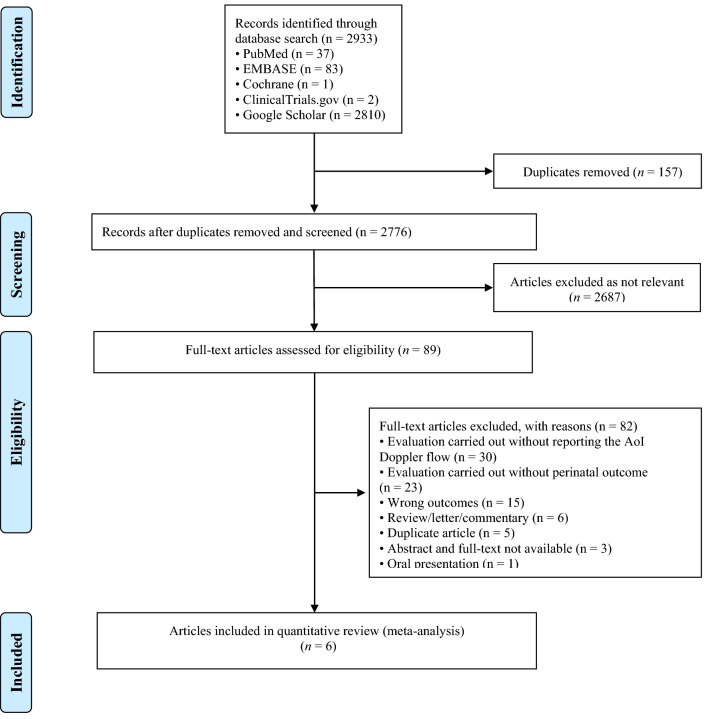
Flow diagram of studies identified in the systematic review

We carried the meta-analysis following the Preferred Reporting Items for Systematic Reviews and Meta-Analysis (PRISMA) statement (www.prisma-statement.org) and the Meta-analysis Of Observational Studies in Epidemiology (MOOSE) statement guidelines [46, 47]. The study protocol was developed before and registered in the International Prospective Register of Systematic Reviews database (PROSPERO ID: CRD42020160983). From the beginning until May 1st 2021, extensive research was conducted using PubMed, EMBASE, the Cochrane Library, ClinicalTrials.gov, and Google Scholar. Constrained words (MeSH in PubMed, Emtree in EMBASE) were combined with free-text keywords (Cochrane Library and Google scholar). The following terms were used as index terms or free-text words: (“fetus” or “fetal”), (“aorta/thoracic” or “isthmus” or “Aortic isthmus”), (“fetal growth retardation” or “Intrauterine growth retardation” or “IUGR” or “Growth retardation”), and (“ultrasonography” or “doppler” or “ultrasound”). Duplicated articles were omitted, and prior reviews were searched for additional articles that met the inclusion criteria [48–50]. Our search strategy is presented in Fig. 1 using a PRISMA diagram. Language restriction was not applied; one of the retrieved articles was in French [35]. We screened for studies comparing anterograde to retrograde aortic isthmus flow in women with FGR in the absence of any documented chromosomal or anatomical abnormalities. The meta-analysis includes studies reporting on the association between AoI Doppler and one of the following adverse perinatal outcomes: perinatal death, stillbirth, neonatal death, severe neonatal morbidity, necrotizing enterocolitis (NEC), sepsis, respiratory distress syndrome (RDS), intraventricular hemorrhage (IVH) (Grade III-IV), bronchopulmonary dysplasia (BPD), adverse neurological outcomes, and admission to a neonatal intensive care unit (NICU). When the same author published multiple papers, only data reporting on the larger study group were included. Review articles and studies reporting on AoI Doppler with no information on the neonatal outcome were excluded.

### Data extraction and quality assessment

Two reviewers (M.L.V and G.R.) reviewed all abstracts and articles independently. We collected complete reports on research that were evaluated potentially acceptable by at least one of the reviewers. The two reviewers (M.L.V. and G.R.) determined whether or not to include the complete reports and extracted data. In case of uncertainty, a third reviewer assessed the manuscript (M.M.). For each included paper, information about the first author, nation, journal, year of publication, setting of recruitment, sample size, and patient characteristics were collected. The AoI flow Doppler (anterograde or retrograde flow) and neonatal outcomes were analyzed. Whenever AoI Doppler results were shown, 2 × 2 tables were extracted. Two reviewers (M.L.V. and G.R.) used the Newcastle–Ottawa scale to evaluate the risk of bias [51].

### Statistical analysis

Meta-analyses were conducted to determine the following adverse perinatal outcomes in singleton pregnancies with growth-restricted fetuses: perinatal death, stillbirth, neonatal death, necrotizing enterocolitis, sepsis, respiratory distress syndrome (RDS), intraventricular hemorrhage (IVH, Grade III-IV), bronchopulmonary dysplasia (BPD), and admission to NICU. These outcomes were chosen by consensus, because they are the most uniformly defined and less influenced by ascertainment bias. According to the Doppler direction of diastolic blood flow, the AoI was categorized as reversed or anterograde. The FGR was defined as an estimated fetal weight (EFW) below the 10th percentile using local reference curves [52, 53]. For each outcome analyzed, the predictive accuracy of anterograde versus retrograde AoI flow was compared. Additionally, this method was utilized to explore heterogeneity. In a conservative approach, the random-effect estimates of event proportion (ES), allowing for variation of true proportion across studies, were considered the ‘main results’, which was calculated using the method of DerSimonian and Laird. To stabilize variances, the pooled estimate was computed using the Freeman-Tukey Double Arcsine Transformation. The exact procedure was used to estimate the confidence interval (CI). Using the I2-statistic, we measured heterogeneity as the proportion of total variance across trials that can be attributed to heterogeneity rather than chance. I^2^-values of 25%, 50%, and 75% corresponded to cut-off criteria for low, moderate, and high degrees of heterogeneity, respectively, in our meta-analysis. All other proportions having a relative confidence interval (CI) were examined in the same manner. RevMan 5.3 (The Nordic Cochrane Centre, 2014, Copenhagen, Denmark) was utilized to extract data and generate forest plots, while Stata 14.1 (Stata corp., College Station, TX, USA) was utilized to analyze bivariate models.

## Results

**Fig. 2:**
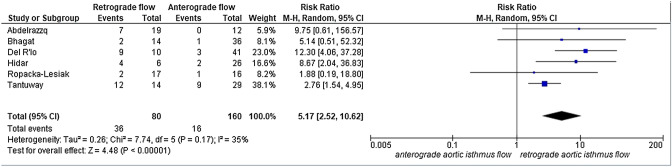
Forest plot of the pooled prevalence of perinatal mortality for all studies included in the meta-analysis

**Fig. 3:**
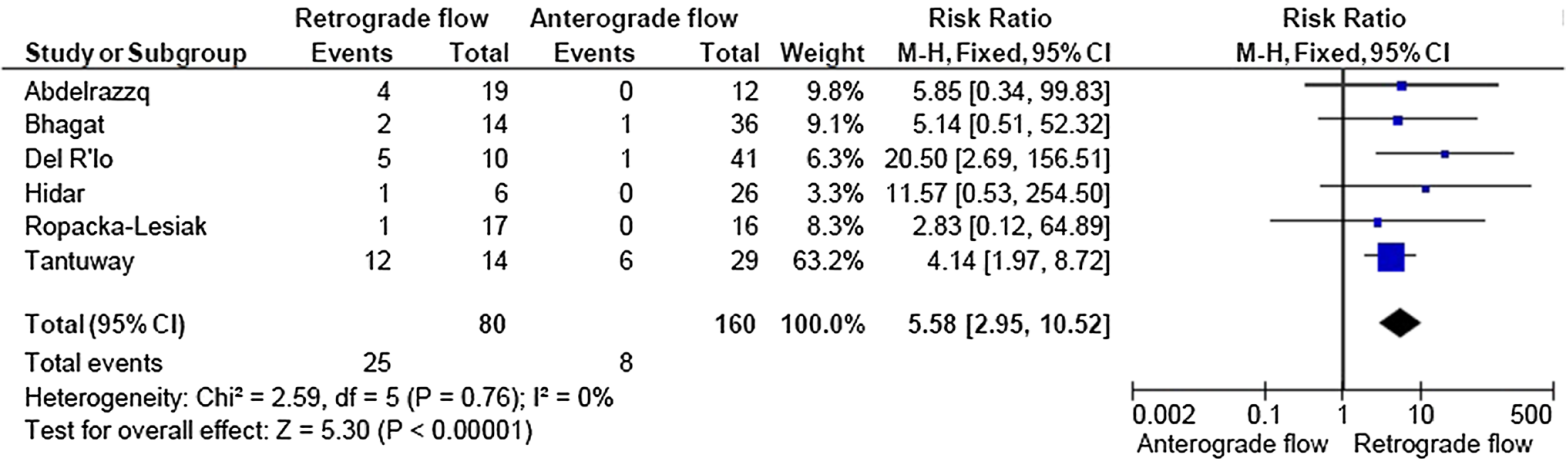
Forest plot of the pooled prevalence of stillbirth for all studies included in the meta-analysis

**Fig. 4:**
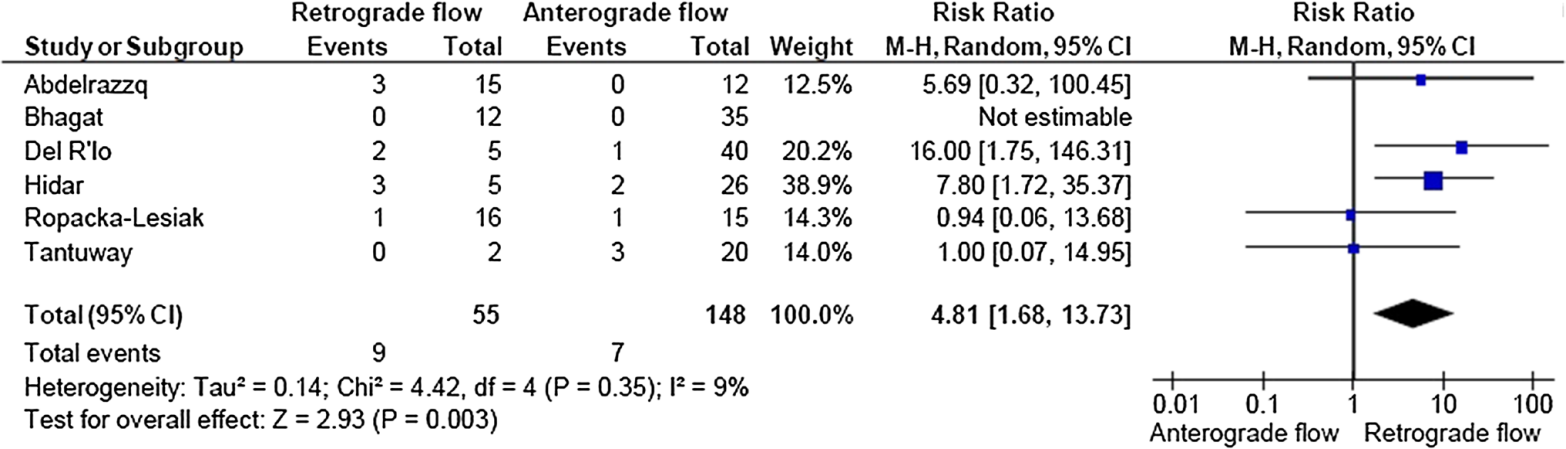
Forest plot of the pooled prevalence of neonatal death for all studies included in the meta-analysis

**Fig. 5:**
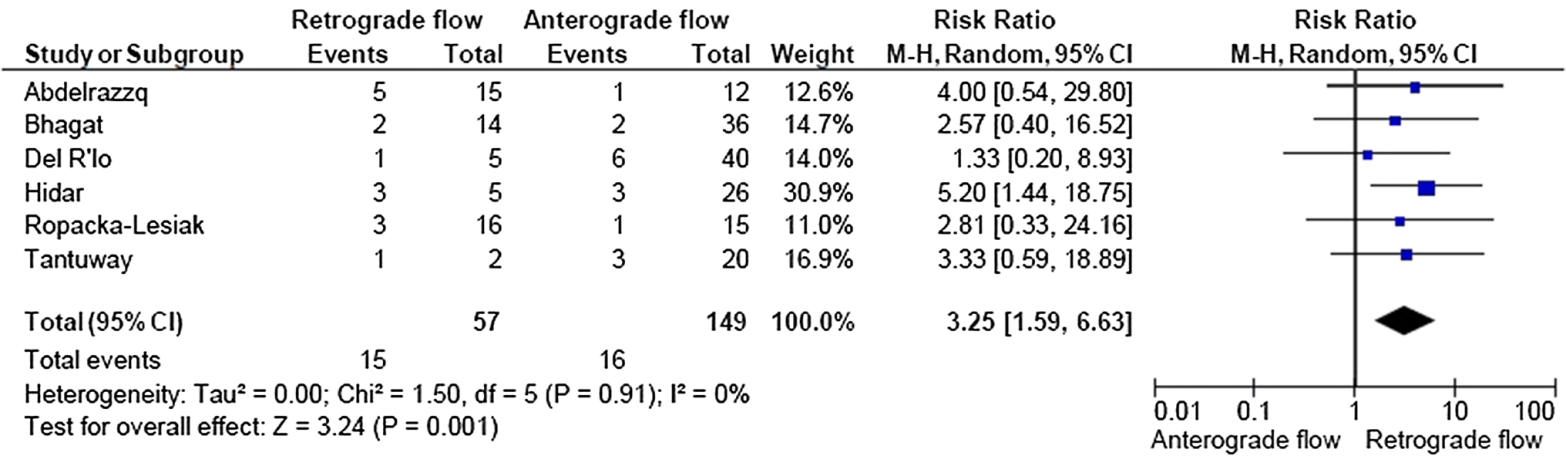
Forest plot of the pooled prevalence of respiratory distress syndrome for all studies included in the meta-analysis

**Fig. 6:**
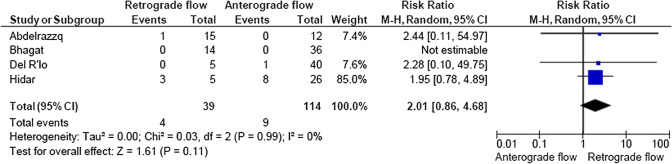
Forest plot of the pooled prevalence of bronchopulmonary dysplasia for all studies included in the meta-analysis

**Fig. 7:**
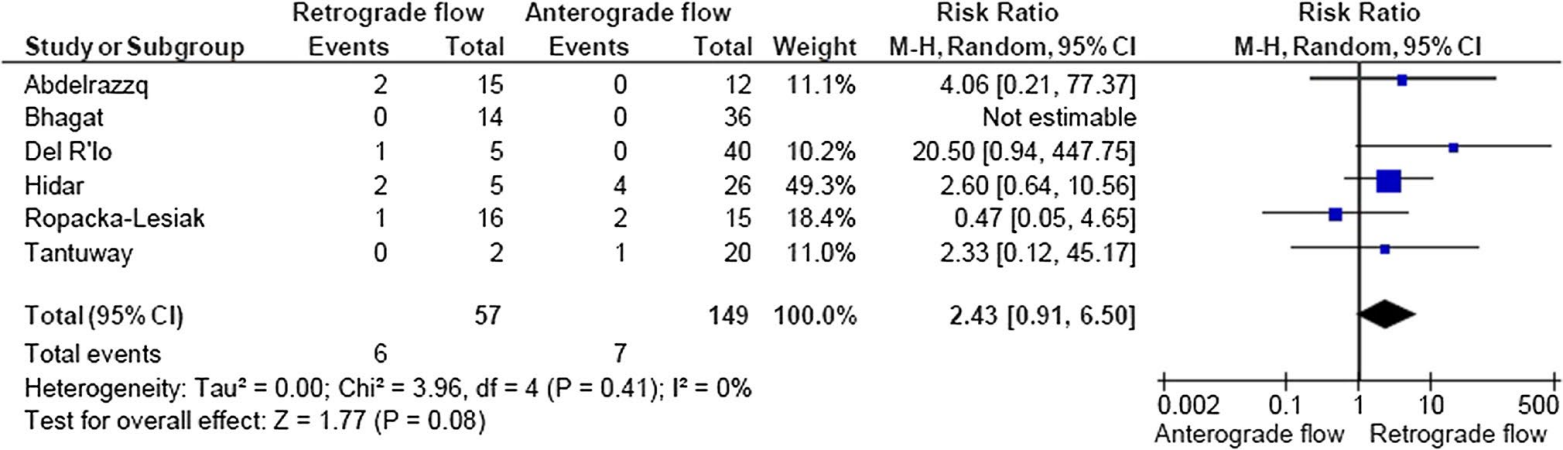
Forest plot of the pooled prevalence of Necrotizing Enterocolitis for all studies included in the meta-analysis

**Fig. 8:**
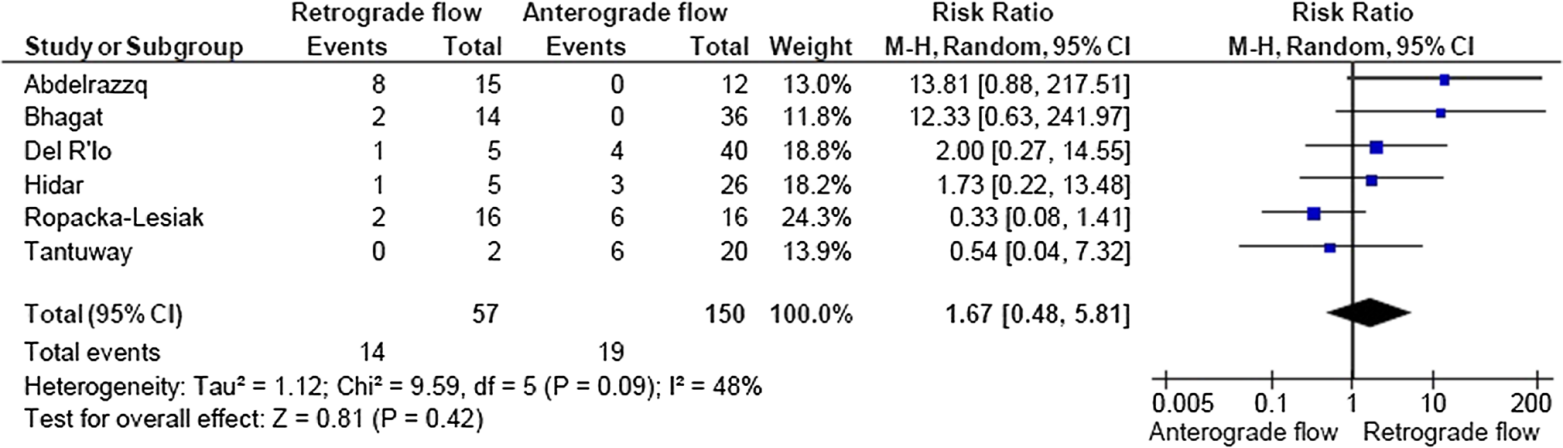
Forest plot of the pooled prevalence of Sepsis for all studies included in the meta-analysis

**Fig. 9:**
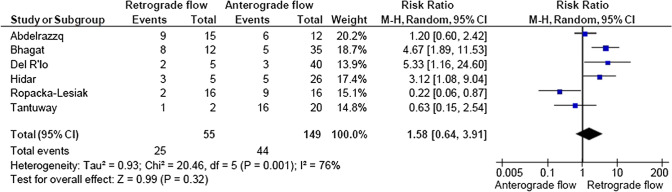
Forest plot of the pooled prevalence of Neonatal Intensive Care Unit for all studies included in the meta-analysis

**Fig. 10:**
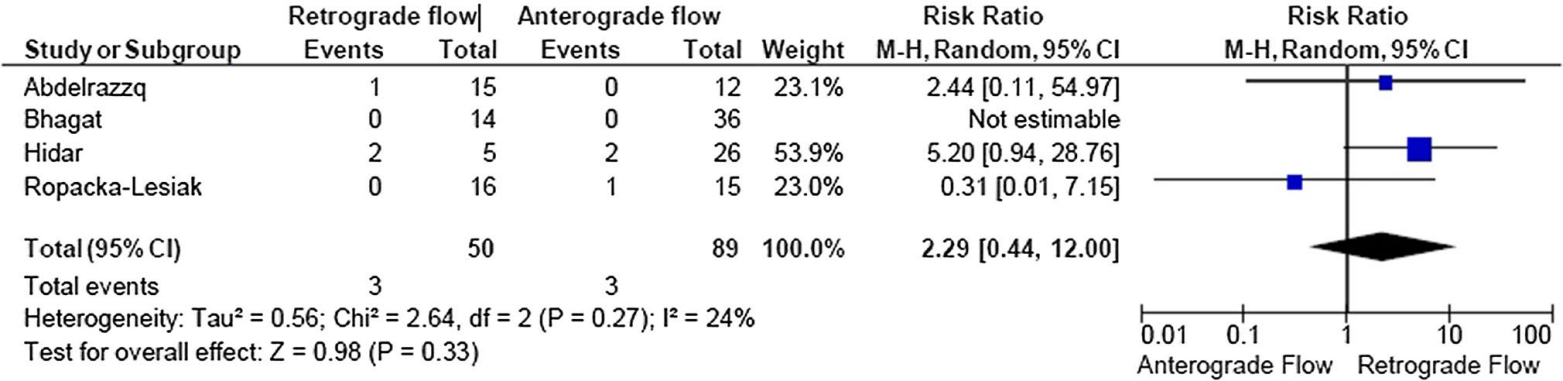
Forest plot of the pooled prevalence of intraventricular hemorrhage, grade III and IV, for all studies included in the meta-analysis

A total of 2933 articles were identified and assessed with respect to their eligibility for inclusion (Fig. 1). After deleting duplicates, we examined the remaining 2813 titles and abstracts, yielding 89 possibly suitable papers. 82 of these were excluded for the reasons described in Fig. 1. As a consequence, 6 studies were included [35–40], with a total of 240 pregnant women (Table 1 and Fig. 1). In five of the six studies, neonatal outcomes were evaluated [36–40]. The missing data were requested to the authors via email, to minimize the risk of bias and to increase the accuracy of the statistical analysis. Table 1 summarizes the features of the studies included. In all the selected studies data were collected prospectively. The trials included 31 to 51 pregnant women. The mean or median age of the mother varied between 25.19 and 32.8 years. All the six studies included FGR fetuses with an EFW below the 10th percentile for gestational age (Table 1). Three studies included FGR with an abnormal umbilical artery Doppler [36, 39, 40], and one study included fetuses with FRG and a cerebroplacental ratio below the 5th centile [38]. Concerning the relationship between neonatal outcomes and anterograde and retrograde AoI Doppler flow, all studies assessed perinatal mortality, intrauterine death, and neonatal death rates. The neonatal outcomes assessed were: RDS, NEC, neonatal sepsis, NICU admission > 14 days. The prevalence of RDS and NEC was reported in six studies, while IVH [35–37, 40] and BPD were reported in four studies [35, 36, 38, 40]. Except for one study [37], all the studies considered late decelerations on the cardiotocography an indication for immediate delivery, whereas US Doppler findings were interpreted in different ways. Del Rio et al. [38] and Hidar et al. [35] evaluated delivery in the context of Doppler parameter decline. Abdelrazzq et al. [36] performed the delivery when reversed “A wave” in the ductus venosus Doppler was observed, while Bhagat et al. [40] considered umbilical artery's reversed flow as indication to delivery. Women with anterograde AoI Doppler flow had a mean or median gestational age of 32.2–37.6 weeks, whereas those with retrograde AoI Doppler flow had a mean or median gestational age of 27.2–35.33 weeks. Tantuway et al. [39] did not report the gestational age at the moment of delivery. Five of the six studies assessed the fetal AoI Doppler using either the longitudinal aortic arch or the three-vessel and trachea views with a 30° insonation angle. While Hidar et al. [35] evaluated the fetal AoI only in the longitudinal aortic arch view [54]. The Newcastle–Ottawa Scale was used to evaluate the quality of the research, and the results revealed an overall high score for the selection and comparability of study groups (five studies out of six), as well as for the determination of the outcomes of interest (Table 2)[51]. RDS, IVH, BPD, NEC, sepsis, and NICU hospitalization > 14 days were studied comparing anterograde and retrograde AoI flow. Table 3 describes all the outcomes analyzed. The overall rate of perinatal mortality and stillbirth were higher in FGR fetuses with AoI retrograde flow, with a risk ratio of 5.17 (2.52–10.62, 95% CI; I^2^ = 35%;  *P* < 0.00001) (Fig. 2) and 5.58 (2.95–10.52, 95% CI; I^2^ 0%; *P* < 0.00001) (Fig. 3), respectively. In the AoI retrograde flow group the risk ratio of neonatal mortality and RDS were 4.81 (1.68–13.73, 95% CI; I^2^ 9%; *P* = 0.003) (Fig. 4). and 3.25 (1.59–6.63, 95%CI; I2 0%; *P* = 0.001), respectively (Fig. 5). For NICU admission and sepsis, the risk ratio in the AoI retrograde flow group were 1.58 and 1.67, respectively (Figs. 6,7,8, 9 and 10).

**Table 1:** Characteristics of six studies included in systematic review and meta-analysis, on prognostic accuracy of the aortic isthmus flow in fetuses with intrauterine growth restriction in the prediction of adverse perinatal outcome

ID	First author, CountRry, Journal, Year	Total AoI N	Anterograde AoI N	Retrograde AoI N	Design	Included	Outcomes	Decision on delivery	GA included in the study	GA at delivery
1	Khalil Abdelrazzq, Turkey, Acta Obstet Gynecol Scand, 2013	31	12	19	Prospective study	Fetal growth restriction (estimated fetal weight < 10th percentile for gestational age) and abnormal umbilical artery Doppler results	Intrauterine death, neonatal death, NICU and complications of preterm birth, such as respiratory distress syndrome (RDS), intraventricular hemorrhage (IVH), bronchopulmonary dysplasia (BPD), necrotizing enterocolitis (NEC) and neonatal sepsis	When the reverse “a wave” at the DV Doppler was seen, or if there was any other fetal distress sign such as late decelerations on the non-stress test	From 24 to 37 weeks of gestation	For Anterograde AoI 35 weeks (range 34–36) For retrograde AoI 32 weeks (range 25–37)
2	Mariola Ropacka-Lesiak, Poland, Ginekol Pol, 2014	33	16	27	Prospective study	Fetal growth restriction (estimated fetal weight < 10th percentile for gestational age)	Intrauterine death, neonatal death, complications of preterm birth, such as respiratory distress syndrome (RDS), intraventricular hemorrhage (IVH), necrotizing enterocolitis (NEC)	NS	From 24 to 37 weeks of gestation	For Anterograde AoI 34.38 ± 3.72 For retrograde AoI 35 ± 3.98
3	M. Del Rio, Spain, ISUOG, 2008	51	41	10	Prospective study	Fetal growth restriction (estimated fetal weight < 10th percentile for gestational age) and cerebroplacental ratio < 5th centile	Intrauterine death, neonatal death, NICU and complications of preterm birth, such as respiratory distress syndrome (RDS), intraventricular hemorrhage (IVH grade is not reported), bronchopulmonary dysplasia (BPD), necrotizing enterocolitis (NEC) and neonatal sepsis	In the presence of deterioriation of Doppler parameters, or in presence of fetal demise or if there was any other fetal distress sign such as late decelerations on the non-stress test or if there were worsening maternal conditions as determined by the managing physician	From 24 to 36 weeks of gestation	For Anterograde AoI 32.2 (24–37) For retrograde AoI 27.2 (25–32.1)
4	Hidar S., Tunisie, J Gynecol Obstet Biol Reprod, 2004	32	26	6	Prospective study	Fetal growth restriction (estimated fetal weight < 10th percentile for gestational age)	Only total perinatal mortality	In the presence of deterioriation of Doppler parameters or if there was any other fetal distress or if there were worsening maternal conditions as determined by the managing physician	From 28 to 38 weeks of gestation	For Anterograde AoI 34.23 ± 2.85 For retrograde AoI 35.33 ± 2.31
5	Tantuway B., India, Int J Reprod Contracept Obstet Gynecol, 2018	43	29	14	Prospective study	Fetal growth restriction (estimated fetal weight < 10th percentile for gestational age) and abnormal umbilical artery Doppler results	Intrauterine death, neonatal death, NICU and complications of preterm birth, such as respiratory distress syndrome (RDS), necrotizing enterocolitis (NEC) and neonatal sepsis	Decision for delivery was taken as per the hospital protocol (not specified)	From 28 to NS weeks of gestation	NS for Anterograde AoI NS for retrograde AoI
6	Bhagat B., India, J Evid Based Med Healthc, 2018	50	36	14	Prospective study	Fetal growth restriction (estimated fetal weight < 10th percentile for gestational age) and abnormal umbilical artery Doppler results (PI > 95° percentile)	Only perinatal death (not specified: Intrauterine death and neonatal death), NICU > 7 days (not 14 days) and complications of preterm birth, such as respiratory distress syndrome (RDS), intraventricular hemorrhage (IVH), bronchopulmonary dysplasia (BPD), necrotizing enterocolitis (NEC) and neonatal sepsis	For uncomplicated IUGR foetuses was through induction of labor at 38–40 GA after maternal steroid application. For complicated IUGR foetuses delivery was considered in cases of abnormal fetal heart rate pattern, reversed flow in the umbilical artery, abnormal biophysical profile	From 28 to 40 weeks of gestation	For Anterograde AoI 37.6 weeks ± 13 days* For retrograde AoI 33 weeks ± 10 days*

*AoI* Aortic Isthmus flow, *NS* not specified, *GA* gestational age, *IUGR* intrauterine Growth Restriction, *NICU* neonatal intensive care unit, *NICU* neonatal unit, *IVH* intraventricular hemorrhage, *NEC* necrotizing enterocolitis, *BPD* bronchopulmonary dysplasia

*Data collected through mail

**Table 2:** Quality assessment of the 6 included studies according to Newcastle–Ottawa Scale

Author	Year	Selection	Comparability	Outcome
Abdelrazzq et al.	2013	★ ★ ★	★	★ ★ ★
Ropacka-Lesiak et al.	2014	★ ★★	★	★ ★
Del Rio et al.	2008	★ ★ ★	★	★ ★ ★
Hidar et al.	2004	★ ★ ★	★	★
Tantuway et al.	2018	★ ★ ★		★ ★★
Bhagat et al.	2018	★ ★ ★	★	★

A study can be receive a maximum of one star for each numbered item within the Selection and Outcome categories

A maximum of two stars can be given for Comparability

**Table 3:** Reported the total number of fetuses with intrauterine retard growth, the number of fetuses with the anterograde and retrograde flow in Aortic Isthmus and the number of adverse perinatal outcomes investigated in the metanalysis

Study	Tot. N	N Anterograde AoI flow	N Retrograde AoI flow	Outcomes																	
				Perinatal death		Stillbirth		Neonatal death		RDS		IVH		BPD		NEC		Sepsis		NICU	
				Ant.	Retr.	Ant.	Retr.	Ant.	Retr.	Ant.	Retr.	Ant.	Retr.	Ant.	Retr.	Ant.	Retr.	Ant.	Retr.	Ant.	Retr.
Abdelrazzq et al. (2013)	31	12	19	0	7	0	4	0	3	1	5	0	1	0	1	0	2	0	8	6	9
Ropacka-Lesiak et al. (2014)	33	16	17	1	2	0	1	1	1	1*	3*	1	0	NS	NS	2	1	6*	2*	9*	7*
Del Rio et al. (2008)	51	41	10	3	9	1	5	1	2	6	1	NS	NS	1	0	0	1	4	1	3	2
Hidar et al. (2004)	32	26	6	4	2	0*	1*	2*	3*	3*	3*	2*	2*	8*	3*	4*	2*	3*	1*	5*	3*
Tantuway et al. (2018)	43	29	14	9	12	6	12	3	0	3	1	NS	NS	NS	NS	1	0	6	0	16	1
Bhagat et al. (2018)	50	36	14	1	2	1*	2*	0*	0*	2	2	0	0	0	0	0	0	0	2	5*	8*

*AoI* Aortic Isthmus flow, *NS* not specified, *RDS* Respiratory distress syndrome, *IVH* Intraventricular hemorrhage (Grade III–IV) , *BPD* Bronchopulmonary dysplasia, *NEC* Necrotizing enterocolitis, *NICU* Neonatal Intensive Care Unite stay > 14

*Data collected through mail

## Discussion

In FGR fetuses, it is critical to establish the optimal time of delivery based on US Doppler examination and other fetal well-being indicators [55]. In the early stage of FGR, increased pulsatility index (PI) in the UA preceded a sequence of Doppler changes, before the occurrence of fetal acidosis [18, 56]. Subsequently, the absence and the reversed EDF in the UA, are expression of a late decline in early FGR fetuses. These findings are finally followed by abnormalities in the ductus venosus, absence or reversed a-wave, indicative of cardiovascular and metabolic failure [57, 58]. Steroids administration for lung maturation, magnesium sulfate prophylaxis for neuroprotection, delivery in a tertiary care center, and close fetal monitoring to define the optimal delivery time, are the only effective interventions to reduce the occurrence of acidosis-related complications in early FGR fetuses [59, 60]. Neonatal complications due to iatrogenic prematurity are even higher [61]. The Trial of Randomized Umbilical and Fetal Flow in Europe (TRUFFLE) revealed that following a strict protocol including DV Doppler assessment and computerized cardiotocography (cCTG) for fetal monitoring and decision making for time of delivery, significantly improves fetal and neonatal outcomes for early FGR fetuses [62]. The management protocol from the TRUFFLE study has been subsequently included in the majority of guidelines and recommendations on the management of FRG fetuses by the main international societies. AoI Doppler assessment was not included in the management protocol used in the TRUFFLE. In the case of late-onset FGR, several guidelines have proposed different management, including fetal surveillance combining a weekly UA Doppler and cCTG, with increased fetal controls if the UA is abnormal [7, 16]. IUSOG guidelines recommend monitoring according to FGR severity and UA Doppler abnormalities [10]. Other studies demonstrated a strong association between MCA Doppler alterations and fetal morbidity and mortality in late-onset FGR after 35 gestational weeks [63]. Low CRP was also related to a greater risk of fetal distress in labor, a lower fetal pH, and an increased risk of caesarean section and neonatal intensive care unit admission in fetuses with late-onset FGR [11, 63–65]. Other guidelines recommend delivery in cases of late-onset FGR with brain sparing [66]. In addition, several studies suggested that changes in the AoI might represent an intermediate step between placental insufficiency-hypoxemia and fetal cardiac failure, occurring prior to DV abnormalities which are associated with fetal cardiovascular deterioration. Using the AoI Doppler, it is possible to estimate the fetal ventricular and cardiovascular hemodynamic status. Anterograde AoI blood flow redirects oxygenated blood to the heart and brain circulations [43], in animal models, it has been proven that when the fetal AoI flow is anterograde, brain oxygenation is preserved [67, 68]. Placental insufficiency is usually associated with an increase in placental vascular resistance. This condition, together with the fetal cerebral vasodilation, can be responsible of the reduction in the anterograde blood flow within the aortic isthmus, and in more severe cases of the retrograde flow. When the blood flow within the aortic isthmus is reversed, the more oxygenated blood coming from the pulmonary artery and the aorta is directed to the placenta, and therefore the brain will be perfused by poorly oxygenated blood, inappropriate for normal neurodevelopment, [43, 67, 69]. Some authors have suggested to include reversed AoI in the management of FGR fetuses, as a sign of severe placental insufficiency that could be useful for decision making on timing of delivery beyond 34 weeks of gestation [12, 13]. However, more data are needed to support this intervention. Our meta-analysis showed that AoI retrograde flow is a reliable predictor of neonatal outcomes. We discovered that an AoI retrograde Doppler flow significantly increased the risk of perinatal death, stillbirth, and RDS. Our meta-analysis represents the most extensive analysis on the AoI Doppler flow in case of FGR, with good heterogeneity in all the outcomes reported. However, there are some limitations to be considered. In terms of comparability and reported outcomes, half of the studies had a risk of bias (Table 2). Secondly, in all the included studies, the delivery timing was established according to local protocols, which may have influenced some of the fetal and neonatal outcomes. Furthermore, because the study populations ranged from early to late severe FGR, these differences may have affected the prevalence of unfavorable perinatal outcomes. Finally, the sub-analysis of preterm birth complications was limited given the small sample of publications that clearly recorded all the neonatal outcomes.

## Conclusion

AoI Doppler assessment may increase the accuracy of the prediction of adverse perinatal outcomes in singleton pregnancies affected by FGR. The findings from this meta-analysis might be taken into account to determine the appropriate time of delivery for fetuses with FGR, despite the limited evidence provided from the available literature. The usefulness of the AoI Doppler for directing clinical management in FGR fetuses needs to be assessed via clinical trials, which are now underway.

### Supplementary Information

Below is the link to the electronic supplementary material.

Supplementary file1 (DOCX 29 KB)

## Data Availability

The data are available.
