# Supplementary material for: Fetal aortic isthmus Doppler assessment to predict the adverse perinatal outcomes associated with fetal growth restriction: systematic review and meta-analysis
Source: Arch Gynecol Obstet. 2023 Apr 19;309(1):79–92. doi: 10.1007/s00404-023-06963-4 (PMC10769912; doi:10.1007/s00404-023-06963-4)
Supplement: Supplementary file 1 — Supplementary file1 (DOCX 29 KB) [file 404_2023_6963_MOESM1_ESM.docx]

**SUPPLEMENTARY Table S1:** Search Strategy.

Search terms for PubMed

| **SET** | **TOPIC** | **SEARCH TERMS** |
| --- | --- | --- |
| 1 | Fetal aortic isthmus (AoI) | "fetus" |
| 2 |  | "fetal" |
| 3 |  | "aorta/thoracic" |
| 4 |  | "isthmus" |
| 5 |  | "Aortic isthmus" |
| 6 | Set 1, 2 were combined with "or" | |
| 7 | Set 3, 4, 5 were combined with "or" | |
| 8 | Set 6, 7 were combined with "and" | |
| 9 | Intrauterine Growth Restriction (IUGR) | "fetal growth retardation" |
| 10 |  | "fetal growth retardation"[MeSH Terms] |
| 11 |  | "fetal" |
| 12 |  | "growth" |
| 13 |  | "retardation" |
| 14 |  | "intrauterine" |
| 15 |  | "growth" |
| 16 |  | "retardation" |
| 17 |  | "intrauterine growth retardation" |
| 18 |  | "Iugr" |
| 19 | Set 11, 12, 13 were combined with "and" |  |
| 20 | Set 14, 15, 16 were combined with "and" |  |
| 21 | Set 10, 18, 9, 19, 17 were combined with "or" |  |
| 22 | Set 10, 19, 9, 18 were combined with "or" |  |
| 23 | Growth retardation | "growth and development"[Subheading] |
| 24 |  | "development" |
| 25 |  | "growth and development" |
| 26 |  | "growth"[MeSH Terms] |
| 27 |  | "retardation" |
| 28 | Set 15, 24 were combined with "and" |  |
| 29 | Set 23, 28, 25, 12, 26 were combined with "or" |  |
| 30 | Set 29, 27 were combined with "and" |  |
| 31 | Set 9, 21, 22, 30 were combined with "or" |  |
| 32 | Diagnostic Procedure | "ultrasonography" |
| 33 |  | "doppler" |
| 34 |  | "diagnostic imaging"[Subheading] |
| 35 |  | "diagnostic" |
| 36 |  | "imaging" |
| 37 |  | "diagnostic imaging" |
| 38 |  | "ultrasound" |
| 39 |  | "ultrasonography"[MeSH Terms] |
| 40 |  | "ultrasonics"[MeSH Terms] |
| 41 |  | "ultrasonics" |
| 42 | Set 35, 36 combined with "and" |  |
| 43 | Set 34, 42, 37, 38, 39, 32, 38, 40, 41 were combined with "or" |  |
| 44 | Set 32, 33, 43 combined with "or" |  |
| 45 | Set 8, 31, 44 were combined with "and" |  |

Search terms for Scopus 72

| **SET** | **TOPIC** | **SEARCH TERMS** |
| --- | --- | --- |
| 1 | Fetal aortic isthmus (AoI) | "fetus" |
| 2 |  | "fetal" |
| 3 |  | "aorta/thoracic" |
| 4 |  | "isthmus" |
| 5 |  | "Aortic isthmus" |
| 6 | Set 1, 2 were combined with "or", | |
| 7 | Set 3, 4, 5 were combined with "or" | |
| 8 | Intrauterine Growth Restriction (IUGR) | "fetal growth retardation" |
| 9 |  | "intrauterine" |
| 10 |  | "growth" |
| 11 |  | "retardation" |
| 12 |  | "iugr" |
| 13 | Set 8, 9, 12, 10 were combined with "or" | |
| 14 | Set 14, 10, 11 were combined with "and" | |
| 15 | Diagnostic Procedure | "ultrasonography" |
| 16 |  | "doppler" |
| 17 |  | "ultrasound" |
| 18 | Set 15, 16, 17 were combined with "or" | |
| 19 | Set 6, 7, 14, 18 were combined with "and" | |

Search terms for EMBASE

| **SET** | **TOPIC** | **SEARCH TERMS** |
| --- | --- | --- |
| 1 | Fetal aortic isthmus (AoI) | "fetus"/exp |
| 2 |  | "fetus" |
| 3 |  | "fetal" |
| 4 |  | "aorta/thoracic"/exp |
| 5 |  | "aorta/thoracic" |
| 6 |  | "isthmus" |
| 7 |  | "Aortic isthmus" |
| 8 | Set 1, 2, 3 were combined with "or" | |
| 9 | Set 4, 5, 6, 7 were combined with "or" | |
| 10 | Set 8, 9 were combined with "and" | |
| 11 | Intrauterine Growth Restriction (IUGR) | "fetal growth retardation" |
| 12 |  | "fetal growth retardation"/exp |
| 13 |  | "fetal" |
| 14 |  | "growth" |
| 15 |  | "retardation" |
| 16 |  | "intrauterine" |
| 17 |  | "growth" |
| 18 |  | 'growth'/exp |
| 19 |  | "retardation" |
| 20 |  | 'retardation'/exp |
| 21 |  | "intrauterine growth retardation"/exp " |
| 22 |  | "intrauterine growth retardation" |
| 23 |  | "iugr" |
| 24 | Set 18, 17 were combined with "or" |  |
| 25 | Set 20, 19 were combined with "or" |  |
| 26 | Set 16, 24, 33 were combined with "and" |  |
| 27 | Growth retardation | "growth and development” |
| 28 |  | "development" |
| 29 |  | "retardation" |
| 30 |  | "retardation"/exp |
| 31 |  | "growth retardation"/exp |
| 32 |  | "growth retardation" |
| 33 | Set 30, 29 were combined with "or" |  |
| 34 | Set 24, 33 were combined with "and" |  |
| 35 | Set 12, 11, 21, 22, 26, 23, 31, 32, 34 were combined with "or" |  |
| 36 | Diagnostic Procedure | "ultrasonography" |
| 37 |  | "doppler" |
| 38 |  | "ultrasonography"/exp |
| 39 |  | "ultrasound" |
| 40 |  | "doppler"/exp |
| 41 |  | "ultrasound/exp |
| 42 | Set 38, 36, 40, 37, 41, 39 were combined with "or" | |
| 43 | Set 8, 9, 35, 42 were combined with "and" | |
